# Trends in the Use of Total Hip Arthroplasty in the Pediatric Population: A Review of the Literature

**DOI:** 10.7759/cureus.43978

**Published:** 2023-08-23

**Authors:** Correggio L Peagler, Alexander J Dobek, Sean Tabaie

**Affiliations:** 1 Orthopaedic Surgery, George Washington University School of Medicine and Health Sciences, Washington DC, USA; 2 Orthopaedic Surgery, Children's National Hospital, Washington DC, USA

**Keywords:** total hip arthroplasty, legg-calve-perthes disease, avascular osteonecrosis, pediatric hip disease, triradiate cartilage, pediatric hip replacement, total hip replacement (thr)

## Abstract

Current literature shows that the utilization of total hip arthroplasty (THA) to address pediatric hip pathology has seen a significant rise worldwide in recent decades. However, performing THA in pediatric patients presents unique challenges due to their increased activity levels, varying skeletal maturity, and diverse medical conditions. These challenges have relegated THA to a secondary option for young patients. Nonetheless, despite these difficulties, recent studies have demonstrated a growing prevalence of THA in pediatrics. Consequently, there is an urgent need for a comprehensive review of the existing literature on this topic. In this study, we examined large database and single-institution studies involving pediatric patients aged 21 years and under who underwent THA. The primary indications for THA in this population were osteoarthritis, osteonecrosis, and inflammatory arthritis. To ensure informed decision-making for pediatric patients, it is crucial to gather consolidated information on trends and outcomes related to THA indications. This review aims to provide insights into these trends and facilitate better decision-making for the treatment of pediatric patients.

## Introduction and background

Total hip arthroplasty (THA) has revolutionized the treatment of degenerative hip diseases in adults, boasting exceptional long-term outcomes and a high success rate. Over the years, advancements in surgical techniques have further improved its efficacy, with a remarkable 58% of THAs estimated to last beyond 25 years, accompanied by a low revision rate of 4.3% within the first decade, as indicated by a 2022 analysis of the National Joint Registry [1]. Encouraged by these achievements, THA has also emerged as a viable option for addressing joint diseases in pediatric patients.

However, the pediatric population presents unique challenges for THA. These young patients exhibit increased activity levels, varying skeletal maturity, and a range of distinct medical conditions, which pose a risk to the long-term survival of implants [2-4]. Consequently, there is limited evidence available on the optimal implant choices and surgical approaches for this population, leading to apprehension among surgeons when considering THA for pediatric patients.

Nevertheless, recent literature examining the trends in pediatric THA indicates a steady rise in its utilization since the late 1990s. Notably, these studies have demonstrated favorable outcomes in the pediatric population, including low revision rates, effective pain relief, and improved hip joint function [4-8]. This review aims to shed light on the use of THA in patients aged 21 years and under, specifically examining the impact of skeletal maturity, disease indications, and surgical approaches on the overall outcomes. By synthesizing the available data, this review seeks to provide valuable insights into the application of THA in pediatric patients, enabling surgeons to make informed decisions regarding implant selection and surgical strategies.

## Review

Skeletal maturity

When weighing the risks of surgery versus the probability of a positive outcome, many surgeons consider the closing of the triradiate cartilage. However, there is limited support for this consideration, particularly in the context of THA. Past literature estimated the closure of the triradiate cartilage at 12 years for girls and 14 years for boys, based on post-mortem studies of the acetabulum [9]. Surgeons have historically used this closure as a marker of skeletal maturity, believing it enhances implant survival in the pediatric population.

Current literature is now questioning whether the closure of the triradiate cartilage truly impacts surgical outcomes or if it is merely an arbitrary measure. A study from a single institution examined 12 patients (13 hips) who underwent THA despite having open triradiate cartilage. Out of these patients, only one experienced loosening of the acetabular component and instability on two separate occasions. No other complications were observed during the average follow-up of 5.5 years [3]. Although the sample size was too small for statistical analysis, the success of THA in patients with open triradiate cartilage is encouraging. However, further research, observation, and follow-up are necessary to aid in surgical decision-making.

Considering the potential lack of skeletal maturity in pediatric patients and the high likelihood of revision surgery in such young individuals, it is crucial for surgeons to take this into account. Optimizing the time between the initial surgery and potential revision is paramount in guiding decision-making for this population [10]. The aforementioned study challenges the notion of the closing of the triradiate cartilage as a definitive skeletal milestone to wait for when considering THA. Earlier intervention could potentially improve a patient's quality of life sooner and minimize the duration of disability.

Indications and outcomes

THA is increasingly performed in the pediatric population to address a range of conditions, such as pediatric hip diseases (33%), osteonecrosis (12-38%), and systemic inflammatory disease (15-27%). Examples of pediatric hip diseases include developmental dysplasia of the hip, congenital hip deformity, and Legg-Calve-Perthes disease [4-8]. However, performing THA in pediatric patients presents unique challenges due to the differing indications and potential complexities of achieving positive outcomes compared to adult patients.

Studies conducted at single institutions have shown avascular necrosis of the femoral head to be the most common indication for THA [2,3]. Conversely, THA for inflammatory arthritis has declined due to the effectiveness of non-operative treatment options. Importantly, most studies have not found a significant difference in the rate of revision among the various indications for THA [4-8].

For instance, a 2016 study investigating ceramic-on-ceramic arthroplasty reported positive outcomes based on patient reports and a 10-year survivability of 90%. However, it concluded that THA should only be considered a last resort [2]. Another study from 2003 evaluated outcomes using the Harris hip score in 11 patients (15 hips) under 21 years old. The results showed improvements in pain, function, and patient satisfaction, with only four hips requiring revision at the time of the study [11].

Similarly, in a retrospective study of THA in patients aged 16 years and younger, all 18 patients (24 hips) experienced significant pain and function improvements, with no revisions reported during the average follow-up of 3.8 years (Table 1) [12]. However, one patient required adductor tendon release due to persistent contracture [12]. A US population analysis demonstrated that the average hospital stay for pediatric THA has decreased from 7.3 days in 2000 to 3.2 days in 2016, with osteonecrosis being the primary indication for surgery [8]. A large database study reported five-year, 10-year, and 15-year survivability rates ranging from 73% to 94%, with aseptic loosening as the most common cause of revision [7].

**Table 1:** Preoperative and postoperative modified Merle d'Aubigné and Postel scores * Pain: Grade 3 (tolerable, permitting limited activity) in 10 hips; Grade 2 (severe on attempting to walk) in 13 hips; and Grade 1 (severe and spontaneous) in two hips - resulting in the child expressing suicidal thoughts due to pain. † Movement: Thirteen hips were functionally nearly ankylosed before total hip arthroplasty (Grade 1). ‡ Walking: Fourteen patients had impaired gait, which was limited in time and distance with or without crutches (Grade 2), and four patients were wheelchair-bound (Grade 1). ** Charnley Grade A (only one hip involved) in 10 patients; Charnley Grade B (both hips involved) in three patients; Charnley Grade C (multiple joints involved and/or significant medical or psychological impairment) in five patients. Table courtesy Van de Velde et al. [12]. Creative Commons License (CC BY-NC 4.0).

	Preoperative	Postoperative
Global score	6.2	17.7	p < 0.0001
*Pain	2.3	6	p < 0.0001
^†^Movement	2.2	5.7	p < 0.0001
^‡^Walking	1.7**	6	p < 0.0001

Long-term survivorship studies have shown varying results, with 10-year survivability ranging from 70% to 86% [4,7]. Patients with developmental dysplasia of the hip had lower implant survival, and younger patients exhibited more stress shielding due to active bone remodeling. Leg length discrepancy and lower Harris hip scores were associated with a higher likelihood of revision [4]. In a 2014 study assessing the survivorship of THA and total knee arthroplasty in pediatrics, the five-year cumulative percent revision was 4.5%, and patients diagnosed with tumors had a significantly higher mortality rate [5]. Another study from 2018 reported a 96% five-year survivorship of implants in 769 THAs [6].

In summary, THA in the pediatric population aims to improve function, reduce pain, and increase ambulation. Short-term results show promise in terms of pain relief, quality of life, and function. However, long-term data from recent studies indicate varying 10-year survivorship rates ranging from 70% to 86% [4,7].

Surgical approach

In the context of THA, surgeons employ various approaches that offer different levels of durability. When performing THA in the pediatric population, surgeons prioritize implant selection and surgical techniques that enhance the longevity of the replacement. Fixation methods and the choice of bearing surfaces in implants have been utilized interchangeably to improve the survivorship of hip replacements. Surgeons commonly attribute the reduction in postoperative complications and improved revision rates to advancements in the materials used for THA.

Fixation

Three main fixation approaches are commonly used in THA: cemented, cementless, and hybrid. Cemented THA involves using bone cement as a medium to enhance the fixation of surgical implants. This reduces the risk of implant loosening and subsequent revision surgery. Surgeons often opt for the cemented approach when dealing with patients who have poor bone quality, as the addition of cement can help compensate for the compromised natural bone and facilitate implant fixation.

Decisions regarding the use of a specific style of fixation in the pediatric population vary per institution. The use of cementless fixation has shown promising results. In a single-institution study looking at the efficacy of THA in the skeletally immature, all 12 patients (13 hips) underwent cementless fixation with screw augmentation of the acetabular component. There was osseointegration in 92% of the hips [3]. In a 2003 study, four of the 17 hips underwent cementless THA. These four cementless hips experienced no complications, while five of the 13 cemented hips experienced aseptic loosening [11]. In another study looking at the use of cementless fixation in patients aged 16 years and younger, all 18 patients (24 hips) experienced full osseointegration of components. At a mean follow-up of 3.8 years, none of the components needed revision [12].

In addition to single-institution studies showing promising results for cementless fixation, larger population studies show cementless fixation to be a viable option in the pediatric population. Using the Norwegian Arthroplasty Register, one study showed that in patients under 20 years of age, 89% of acetabular components and 95% of the femoral stems were uncemented implants. The 10-year survival of the primary implant, acetabular component, and femoral stem was 70%, 78%, and 90%, respectively [4].

**Figure 1:**
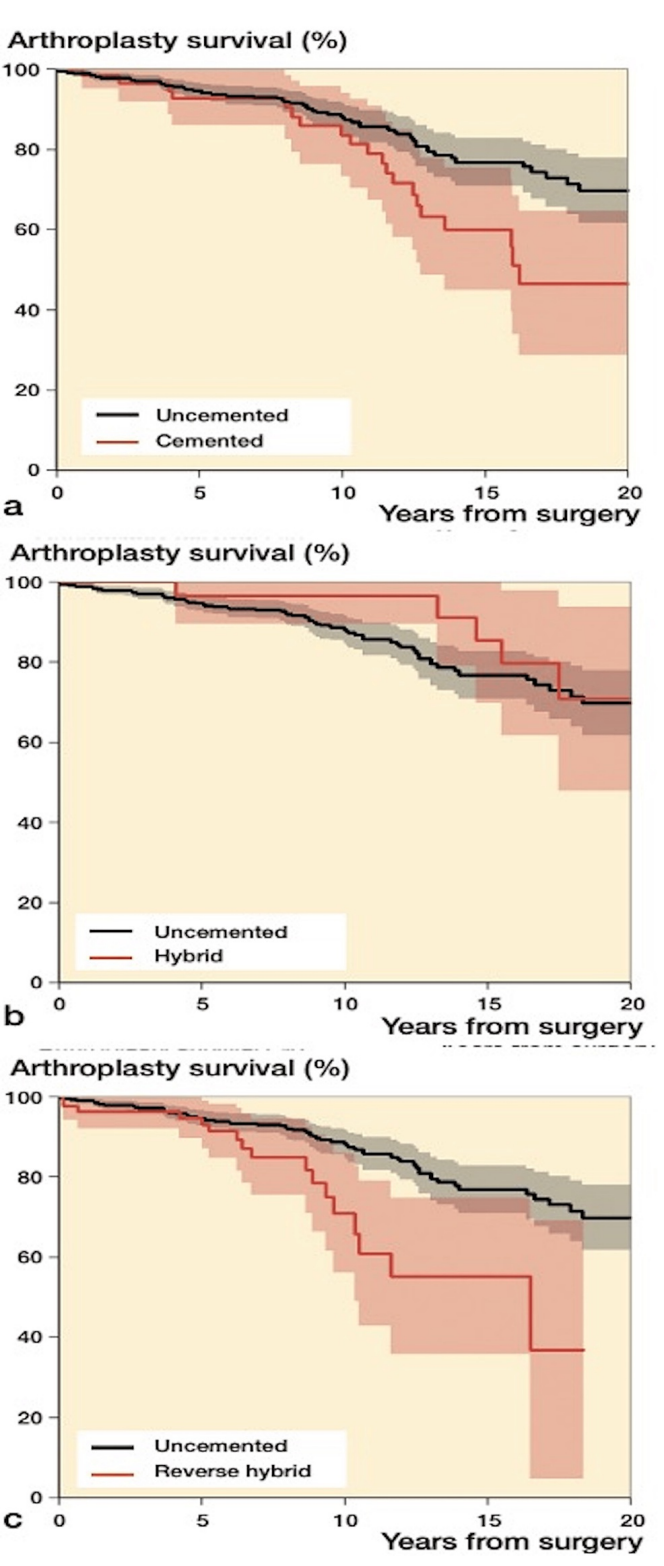
Kaplan–Meier unadjusted survival curves with confidence intervals (shaded areas) for different fixation methods with uncemented fixation as reference (a) Uncemented versus cemented; (b) uncemented versus hybrid; (c) uncemented versus reverse hybrid. Graphs courtesy Halvorsen et al. [7]. Creative Commons License (CC BY 4.0).

The increased survivability of the uncemented femoral stem in the previously mentioned study suggests that a reverse hybrid style fixation could also be a viable choice. On the contrary, using the Nordic Arthroplasty Register Association, it was seen that the reverse hybrid fixation was at higher risk for revision (Figure 1) [7]. Conclusions drawn from this study indicated that cement and cementless fixation are both acceptable for use in pediatrics; however, implant survival is lower than that of older adults. Moreover, using the National Joint Registry for England, Wales, Northern Ireland, and the Isle of Man, it was concluded that there was no statistically significant difference in survivability based on the style of fixation used [6].

In summary, the literature suggests that there is no perceived benefit from using one style of fixation over the other. Therefore, surgeons should take into consideration the quality of bone and the potential for future revision as the pediatric patient ages when choosing an approach to fixation.

Bearing Surface

When performing THA, new bearing surfaces are added to the articulating surfaces of the hip joint, each with its own level of friction. The various types of bearing surfaces include ceramic on ceramic, metal on metal, ceramic on polyethylene, and metal on polyethylene. In a Norwegian Arthroplasty Registry study, researchers reported that surgeons used a variety of bearing surfaces without preexisting support for use. They recommended that surgeons use bearing surfaces that had preexisting support due to the increased need for survivorship of implants in the pediatric population [4]. The primary goal of THA is to achieve optimal survivorship of the replacement joint, and minimizing wear at the bearing surface is crucial due to the fact that an extremely young patient will likely need a revision during their lifetime. By reducing wear and tear, the lifespan of the replaced joint can be prolonged, thereby delaying the need for revision surgery in pediatric patients.

While surgeons understand the importance of choosing the most suitable bearing surface for the pediatric population, there is currently no general consensus on which bearing surface to use. A study looking at 881 hip arthroplasties from 1995 to 2016 depicts this by reporting that 341 replacements were cross-linked polyethylene in the articulation (206 metal on cross-linked polyethylene and 135 ceramic on cross-linked polyethylene), 145 replacements were metal on metal, one was metal on ceramic, 97 were ceramic on ceramic, 78 were polyethylene on metal, 54 were polyethylene on ceramic, and 165 were unidentified [7]. A single-institution study looking at the use of THA in patients under 21 contributed to the conversation by reporting that increased wear was associated with increased loosening of components and that metal on metal, ceramic on ceramic, and cross-linked polyethylene bearing surfaces may decrease wear of bearing surfaces [11].

Regarding ceramic on ceramic-bearing surfaces, a 2015 study looked at the efficacy of using ceramic on ceramic-bearing surfaces in 83 of the 91 patients in the cohort. They found that in this group, the 10-year survival rate was 90.3% (95% CI = 82.4%-98.9%) [2]. In another study that looked at replacements between 2003 and 2017, it was seen that metal on metal and metal on polyethylene-bearing surfaces had lower implant survival when compared to ceramic on ceramic and ceramic on polyethylene (p-value = 0.002) [6]. Conversely, in a single-institution study of patients 16 years and younger undergoing THA, researchers expressed concern about using small, thin ceramic surfaces in the active, very young patient due to potential fracturing of the material and used metal on polyethylene surface to minimize the teratogenic impact on fertile patients [12].

Additionally, cross-linked polyethylene, a modified form of polyethylene, is specifically designed to reduce wear. Rainer et al. found the introduction of this bearing surface to be so important that when they investigated the lower age limit where negative outcomes may outweigh the benefits of THA, patients who underwent surgery prior to 2000, before the introduction of highly cross-linked polyethylene into common practice, were excluded from the study [3]. Similarly, a vast majority of literature acknowledges the introduction of highly cross-linked polyethylene as a milestone for THA in the pediatric population due to its minimal wear of bearing surfaces [2,3,7,8].

It can be concluded from these studies that due to the age and increased activity level of the pediatric patient, the bearing surface chosen should have the most optimal survivorship. It has been shown that ceramic on polyethylene and ceramic on ceramic are both viable options in pediatric patients [2,5,6,11,13-15]. Moreover, cross-linked polyethylene has the potential to be beneficial; however, more research needs to be done to assess its long-term efficacy in the pediatric population.

## Conclusions

The current literature on THA in the pediatric population reveals gaps in research, particularly in patients aged 21 years and younger. Data-driven decision-making is increasingly in demand as THA usage rises. Single-institution studies offer insights for future larger studies, but long-term follow-up data are critical for definitive conclusions, especially concerning implant longevity. Some studies suggest that waiting for triradiate cartilage closure may not be necessary, but further data are needed for a confident determination. Indications for surgery may not significantly impact implant survivorship, except for developmental dysplasia of the hip and tumors, which show an increased risk of revision and higher mortality rates. Regarding surgical approach, fixation style does not significantly affect implant survivorship, with ceramic or metal on polyethylene being the most successful articulations. However, cross-linked polyethylene in the pediatric population lacks specific literature, warranting investigation for long-term survivorship (10-15 years). Future research should explore the impact of skeletal maturity on THA outcomes, indications for THA, and long-term performance of cross-linked polyethylene in patients aged 21 years and younger to improve decision-making and outcomes in pediatric THA.
